# Exploring the relationship between co-abundance of gut microbiota and novel metabolic pathways in different subtypes of irritable bowel syndrome: insights from the American Gut Project

**DOI:** 10.3389/fmed.2025.1615717

**Published:** 2025-07-22

**Authors:** Li-li Han, Chun-feng Mei, Hong Xue

**Affiliations:** Digestive Laboratory of Traditional Chinese Medicine Research Institute of Spleen and Stomach Diseases, Xi-yuan Hospital, China Academy of Chinese Medical Sciences, Beijing, China

**Keywords:** co-abundance group, irritable bowel syndrome subtypes, American Gut Project, gut microbiota, metabolic pathways

## Abstract

**Background:**

Irritable bowel syndrome (IBS) is a prevalent functional gastrointestinal disorder with an unclear etiology. Recent studies have underscored the association between alterations in the gut microbiome and the pathogenesis of IBS. However, limited knowledge exists regarding the co-abundance patterns of gut microbiota and metabolic pathways across different IBS subtypes.

**Methods:**

In this study, we utilized the comprehensive gut microbiome data from the American Gut Project (AGP). Through Spearman correlation analysis, the random forest model, SHAP analysis, and the PICRUSt2 prediction function, we constructed and screened the gut microbiota co-abundance groups and their metabolic characteristics of three cohorts of patients with different subtypes among cohorts of patients with three distinct IBS subtypes: predominant constipation (IBS-C), predominant diarrhea (IBS-D), and unclassified (IBS-U), as well as three non-IBS control groups (non-IBS1, non-IBS2, and non-IBS3, respectively).

**Results:**

Our study findings indicate that, in comparison to their respective non-IBS groups, there was a significant difference in the prevalence of 37.5% specific co-abundance groups (CAGs) identified across all three IBS subtypes: IBS-C, IBS-D, and IBS-U. In addition, the random forest model shows that there are 2–4 characteristic CAGs for each subtype. We also analyzed the co-abundance networks between each CAG and metabolic pathways. Additionally, we analyzed the co-abundance networks between each CAG and metabolic pathways. No significant species-metabolic pathway co-abundance groups were found in the IBS-C group. In the IBS-D group, 50% of CAGs showed significantly different co-abundance with related metabolic pathways compared to the non-IBS control groups, while in the IBS-U group, this figure was 80%. Through the analysis of differentially expressed metabolic pathways, we revealed significant disturbances in SCFAs and LPS metabolic pathways (particularly a marked increase in acetate) in IBS-D patients, whereas IBS-U patients only exhibited a non-significant downward trend in tryptophan metabolic pathways.

**Conclusion:**

These results indicate that the alterations in the gut microbiota and their associated metabolic pathways differ among IBS subtypes, leading to distinct developments and symptoms. This expands our current understanding of the gut microbiota in different IBS subtypes and provides a theoretical foundation for further research.

## 1 Introduction

Irritable bowel syndrome (IBS) is a common functional gastrointestinal disorder characterized by chronic abdominal pain, abnormal bowel function, and bloating. Existing data suggests that IBS is one of the most common gastrointestinal diseases globally, affecting approximately 4% of the population (1). According to the Rome IV criteria, there are four subtypes of IBS with predominant constipation (IBS-C), IBS with predominant diarrhea (IBS-D), IBS with mixed bowel habits (IBS-M), and unclassified IBS (IBS-U) (2). Although the exact pathogenesis is not fully understood, increasing evidence indicates that alterations in the gut microbiota may be closely associated with the onset and development of IBS (3, 4). The gut microbiota constitutes a complex ecosystem populated by trillions of microorganisms that engage in intricate ecological interactions to exchange or compete for nutrients, signaling molecules, and immune evasion strategies. These interactions significantly influence the modulation of immune function, the maintenance of metabolic balance, and the preservation of intestinal barrier integrity (5). Co-abundance refers to the phenomenon where two or more microbes display similar patterns of abundance fluctuations within a specific temporal and spatial scale in an ecosystem. Recent study (6) has shown that distinct subtypes of IBS exhibit variations in their gut microbiota composition. However, the co-abundance of gut microbiota across different IBS subtypes remains under-researched. To address this gap, a proposed methodology involves constructing correlation-based co-abundance networks, which can elucidate the levels of correlation or co-enrichment among the gut microbiota.

The metabolism of intestinal microbiota is diverse and complex. Specific microbial populations may influence IBS symptoms by modulating metabolic pathways. These microbes metabolize nutrients (7) from food, including carbohydrates, proteins, and fats, and produce metabolites such as short-chain fatty acids (SCFAs) (e.g., butyric acid, propionic acid, and acetic acid) (8), which can influence intestinal mucosal health and immune regulation. Conversely, the metabolic activity of the microbiota may lead to the accumulation of toxic metabolites in the intestinal environment, such as ammonia (9), histamine (10), and hydrogen sulfide (11), which may stimulate the intestinal mucosa and exacerbate symptoms. We hypothesize that as the gut microbiota evolves, its metabolic pathways may undergo corresponding alterations, and the variations in metabolic byproducts could potentially underlie the diverse symptomatology of diseases. Therefore, investigating the co-abundance relationships between the gut microbiota and their metabolic pathways in IBS is crucial for a deeper understanding of the etiology of IBS symptoms.

In this study, we aimed to deepen our understanding of the intricate relationships among co-abundance of gut microbiota, IBS subtypes, and metabolic pathways by analyzing sequencing data from the American Gut Project (AGP) using a one-to-one pairing algorithm with an equal number of matched controls. These results highlight the heterogeneity of the gut microbiota and its role in the pathophysiology of IBS, offering new insights and guidance for further mechanistic studies on the interactions of gut microbiota across various IBS subtypes.

## 2 Materials and methods

### 2.1 Data availability

This research utilized the publicly accessible database of gut microbial samples from the American Gut Project, established in November 2012 by the American Gut Consortium (12). Internationally collected samples were initially shipped to local collection points, stored at −80°C, and subsequently transported to the United States. These samples underwent 16S rRNA sequencing (V4 region) using platforms such as the Illumina MiSeq, Illumina HiSeq Rapid Run, and Illumina HiSeq High-Output. The raw fastq data files were obtained from the European Bioinformatics Institute (EBI) database, listed under project ID PRJEB11419. Consent was obtained from all participants following protocols approved by Institutional Review Boards, either from the University of Colorado Boulder (Protocol No. 12-0582; from December 2012 to March 2015) or the University of California, San Diego (Protocol No. 141853; from February 2015 onwards). No personally identifiable information was included or accessed within the public database or this study (12).

### 2.2 Building of fully paired cohorts for IBS and non-IBS controls

This study enrolled a total of 966 self-reported IBS cases and 966 self-reported non-IBS controls. Following established criteria from previous studies (13), we included both IBS and non-IBS participants while excluding individuals who: (1) were aged <18 or >80 years, (2) had BMI <12.5 or >40, (3) resided outside the United States, United Kingdom, or Canada, (4) failed to provide fecal samples, (5) lacked bowel movement quality data, (6) had taken antibiotics within 6 months prior to enrollment, or (7) had type 2 diabetes. Control subjects were selected from a pool of non-IBS candidates using identical exclusion criteria.

The exact matching algorithm was designed based on previously reported microbiota-associated confounding variables, including age, BMI, height, weight, sex, geographical location, alcohol consumption frequency, and dietary intake of meat/eggs, dairy products, vegetables, whole grains, and salted snacks (13). Pairwise Euclidean distances were calculated between IBS patients and non-IBS controls using the aforementioned matching variables, (all of which were centered and scaled to a mean of 0 and variance of 1). One-to-one matching was subsequently performed using open-source R code.^1^

Through the Rome IV diagnostic criteria, we systematically classified and defined the subtypes of IBS. By integrating professional medical evaluations (including assessments by physicians and physician assistants) with comprehensive analysis of patients’ predominant symptoms, bowel movement characteristics, and associated clinical manifestations, we ultimately enrolled study samples representing three distinct IBS subtypes: constipation-predominant (IBS-C), diarrhea-predominant (IBS-D), and unclassified (IBS-U). Specifically, IBS-C was defined by the statement, “I tend to be constipated (have difficulty passing stool).” IBS-D was defined by “I tend to have diarrhea (watery stool).” Due to insufficiently precise and clear data to meet the criteria for IBS-M (mixed bowel habits)—where more than 25% of bowel movements are classified as Bristol Stool Scale (BSS) types 1 or 2 and more than 25% as BSS types 6 or 7—this subtype was excluded from our analysis. IBS-U was defined as a distinct category in this study, comprising subjects who reported normal stool consistency (“I tend to have normal stool shape”). Finally, three sub-cohorts were constructed: IBS-C, IBS-D, and IBS-U, each matched with corresponding non-IBS controls.

### 2.3 Processing of 16S rRNA sequence data

We downloaded the raw fastq files of AGP database from the National Center for Biotechnology Information (NCBI)^2^ and performed data processing using the QIIME2 (14) on the Linux platform. A considerable number of low-abundance features may increase computational demands and potentially affect FDR-corrected *p*-values when comparing variations in high-abundance outcomes, leading to potential false negatives. To prevent such errors, we excluded sequences from the feature table that were present in fewer than 30 samples and had an absolute abundance of less than 10 reads. This resulted in the generation of an Amplicon Sequence Variant (ASV) table for the 16S rRNA sequences. Then use the trained classifier “classifier_g9_13_8_99_V4” for species annotation. Classification was performed on the V4 region of the bacterial 16S rRNA gene, enabling identification of the taxa at the levels of phylum, class, order, family, and genera in the feature table. Further, we employed PICRUSt2 (version alpha.2) (15) to predict functional pathways using the MetaCyc database.^3^

### 2.4 Calculation and analysis of microbiota co-abundance

To investigate the co-abundance of gut microbiota and their correlation with metabolic pathways in different subtypes of IBS, we employed the following computational and analytical methods: we utilized 16S rRNA sequencing data from the AGP database, conducted quality control and denoising on the raw sequencing data to obtain high-quality sequences, and established ASVs based on these sequences. We aggregated similar sequences into genera using clustering algorithms. We selected genera present in at least 20% of the samples with an average relative abundance ≥0.05%, resulting in 37 genera in the IBS-C group, 44 genera in the IBS-D group, and 40 genera in the IBS-U group. We calculated the co-abundance of the microbial community using the relative abundances of these genera across all samples. Using R version 3.4.2, we computed Spearman correlation coefficients for the gut bacteria in the IBS subgroups and the non-IBS (control) group, applying the Benjamini–Hochberg multiple comparison correction method to adjust the *p*-values. We selected co-abundant relationships with the absolute value of correlation coefficients >0.4 for further analysis. We employed the Ward clustering algorithm in R to cluster the bacteria within each co-abundance group (CAG) into eight CAGs, each containing 2–10 genera. Finally, we utilized Cytoscape for visualizing the co-abundance networks.

Next, we calculated the mean relative abundance of each CAG containing bacteria in each subtype, and conducted analysis of Wilcoxon rank-sum test to determine significant differences between different subtypes and the non-IBS control groups. Wilcoxon rank-sum test was used to compare the relative abundance of bacteria within the eight clustered co-abundance groups in each subtype between patients and control groups, followed by statistical result visualization using GraphPad Prism 8.3.0.

### 2.5 Apply random forest and SHAP analysis to screen characteristic gut microbiota CAGs for IBS subtypes

This study used the Random Forest algorithm to screen characteristic CAGs for different IBS subtypes. First, we summed the relative abundances of all CAGs in each subtype to create a new feature matrix. Then, we used stratified sampling to divide samples into a 70% training set and a 30% test set, ensuring equal sample sizes for the IBS and control groups. We built a classification model with the random Forest package, using 500 decision trees. Feature importance was calculated by the mean decrease Gini method and visualized. After 10-fold cross-validation for model stability, we ranked feature importance scores in descending order. After the model training is completed, use the prediction function to predict the categories of the test-set samples, and calculate metrics such as accuracy, sensitivity, and specificity through the confusion matrix. The pROC package was used to plot ROC curves and calculate AUC for discriminative evaluation.

Finally, we used the SHAP method from the iml package to analyze key CAG contributions. To balance efficiency and reliability, we randomly selected 100 test samples for SHAP value calculation (sample size = 50). We compared the contributions of the top six CAGs in IBS subtypes using grouped box plots, visualized with ggplot2. All analyses were conducted in the R 4.3.3 environment.

### 2.6 Analysis of the correlation between bacteria and metabolic pathways

In this study, we specifically focused on the co-abundance relationships between gut microbiota and metabolic pathways across different IBS subtypes. To determine whether the elevation or reduction of certain bacteria may lead to changes in specific metabolic pathways, we utilized PICRUSt2 to map the obtained bacterial abundance data onto MetaCyc pathways. This enabled us to obtain relative abundance data for each pathway and further analyze the correlation between bacterial abundance data and MetaCyc pathway abundance data. Spearman correlation coefficients were calculated using R version 3.4.2, and Cytoscape 3.9.1 software (16) was used for visualizing the co-abundance networks between bacterial abundance and MetaCyc pathway abundance data. We grouped metabolic pathways belonging to the same hierarchical classification together and performed Wilcoxon rank-sum tests on each pathway associated with each CAG in the IBS subtypes group and non-IBS control groups.

## 3 Results

### 3.1 Cohort characteristics

**Figure 1:**
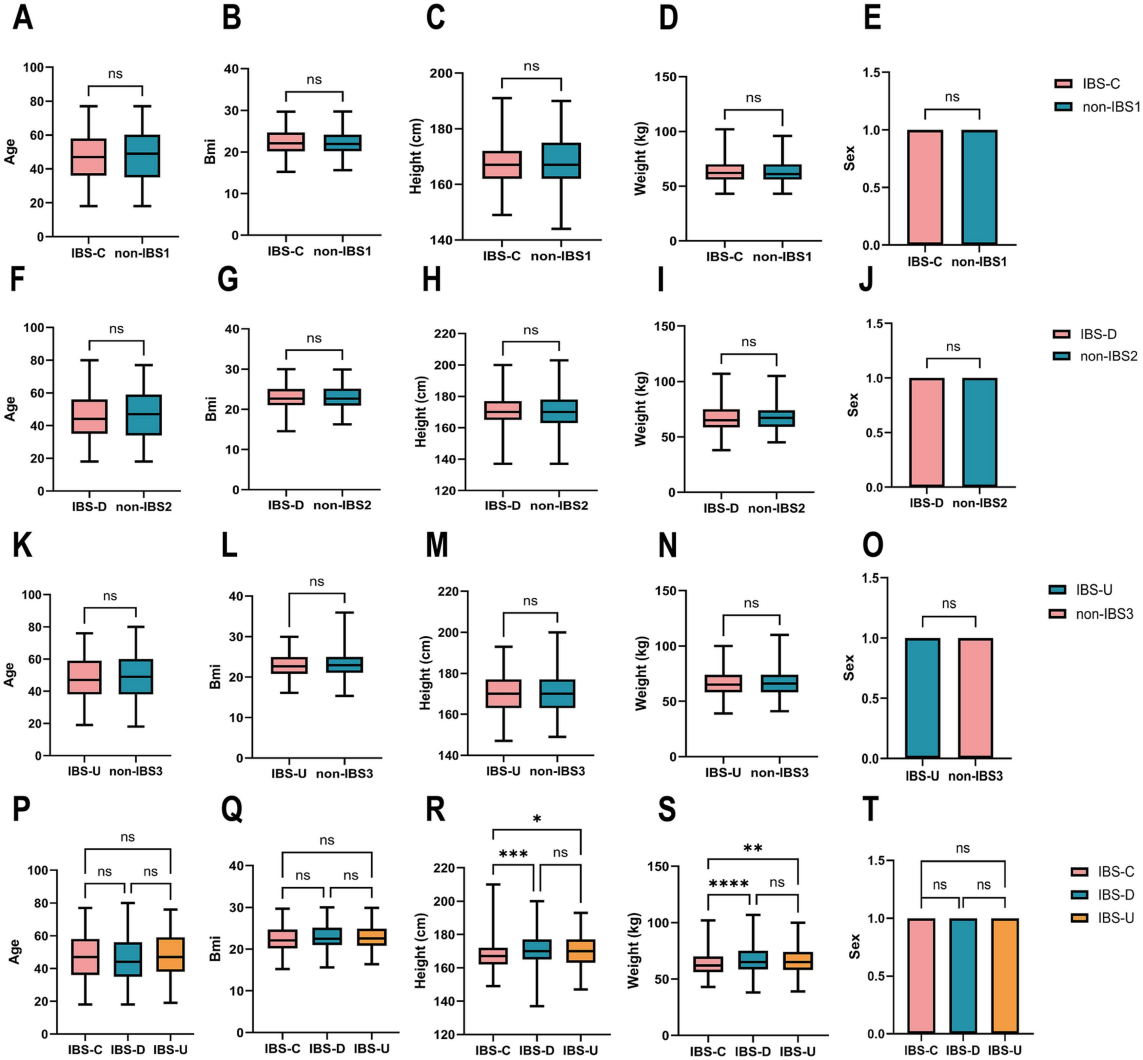
Cohort construction characteristics of IBS subtypes and non-IBS controls. **(A–O)** The paired *t*-test results for age, BMI, height, weight and sex between the three IBS subtypes and non-IBS controls showed no statistically significant difference. **(P–T)** The ordinary one-way ANOVA results for age, BMI, height, weight, and sex between the three IBS subtypes groups. Sex was coded as 1 (male) and 0 (female) for statistical analysis. ^*^p < 0.05, ^**^p < 0.01, ^***^p < 0.001, and ^****^p < 0.0001, ns, not significant.

To investigate the co-abundance of gut microbiota and the potential differences in metabolic pathways among different subtypes of IBS. By considering the patient’s primary symptoms as diagnosed by a medical professional (doctor, physician assistant), bowel movement characteristics, and associated symptoms, we included samples from three subtypes of IBS: IBS-C, IBS-D, and IBS-U, totaling 1,031 pairs. We conducted data screening and preprocessing using the AGP database to obtain high-quality samples from 966 IBS patients, including 230 IBS-C, 365 IBS-D, and 371 IBS-U cases, along with 966 non-IBS pairs matched to each sample, including 230 non-IBS1, 365 non-IBS2 and 371 non-IBS3 cases. There were no differences in age, BMI, height, weight and sex between the IBS-C patients group and the non-IBS1 group (Figures 1A–E), between the IBS-D patients group and the non-IBS2 group (Figures 1F–J), and between the IBS-U patients group and the non-IBS3 group (Figures 1K–O). Meanwhile, no statistically significant differences were observed in age, BMI, or sex among the three IBS subtypes (Figures 1P,Q,T). However, significant differences in height and weight were found between IBS-C and both IBS-D and IBS-U (Figures 1R,S) (*p* < 0.05). To date, no studies have identified height as a factor influencing IBS clinical symptoms. The observed increase in weight aligned with height trends is physiologically expected, given that BMI did not differ significantly among the groups.

### 3.2 The key findings on the co-abundance of gut microbiota in the IBS-C subtype

**Figure 2:**
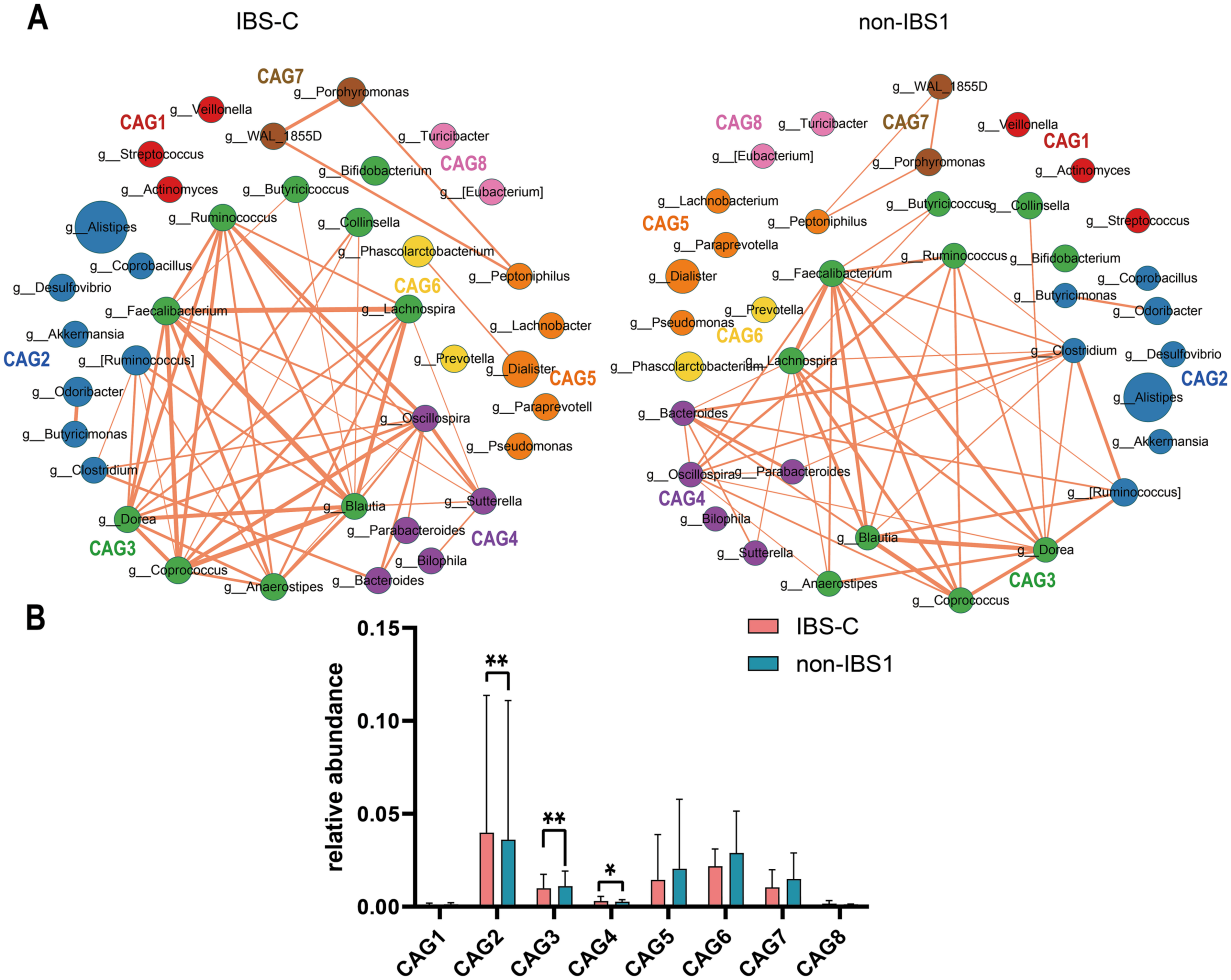
The species co-abundance network and the Wilcoxon rank-sum test results for the average relative abundance of eight CAGs in the IBS-C group. **(A)** The genus-level co-abundance network is presented. The node size represents the average abundance of each genus, and different colors indicate different CAGs. The lines between nodes represent their correlations, with thicker lines indicating larger correlation coefficients. Only positive correlations with coefficients greater than 0.4 are shown in the graph. **(B)** The Wilcoxon rank-sum test results for the average relative abundance of the eight CAGs, with the red color representing the IBS-C group and the pinkish blue color representing the non-IBS1 group. The results not reaching significance were not labeled with “ns” in the figure. ^*^p < 0.05, ^**^p < 0.01, ^***^p < 0.001, and ^****^p < 0.0001.

To investigate co-abundance differences among bacterial genera at the genus level in the IBS-C subtype, we identified 37 enriched bacterial genera categorized into eight CAGs, each containing 2–10 genera (*p* < 0.05). A total of 106 and 98 co-abundances were identified in the IBS-C and non-IBS1 groups, respectively, both exhibiting positive correlations. Compared to the non-IBS1 group, the IBS-C group showed a significant decrease in correlations involving CAG2, CAG3, and CAG4, encompassing genera such as *Clostridium*, *Bacteroides*, *Blautia*, *Ruminococcus*, *Dorea*, *Faecalibacterium*, *Lachnospira*, and *Parabacteroides* (*p* < 0.05). This decrease was particularly pronounced in the *Clostridium* genus within CAG2 and *Bacteroides* within CAG4 (Figure 2A). This may indicate a notable alteration in the gut microbiota structure of IBS-C patients, especially impacting the *Clostridium* and *Bacteroides* genera.

Figure 2B presents the results of the Wilcoxon rank-sum test for the average relative abundances of eight CAGs in IBS-C (*p* < 0.05). The findings reveal that, compared to the non-IBS1 group, the relative abundances of CAG2 (*p* < 0.01) and CAG4 (*p* < 0.05) significantly increased in IBS-C patients, while the average relative abundance of CAG3 significantly decreased (*p* < 0.01) (Figure 2B). These three CAGs are predominantly composed of beneficial bacteria as reported in the literature, including *Bacteroides*, *Akkermansia*, *Ruminococcus*, *Alistipes*, *Butyricimonas*, *Clostridium*, *Faecalibacterium*, and *Blautia*. These observations may represent a novel microbial configuration in IBS-C.

### 3.3 The key findings on the co-abundance of gut microbiota in the IBS-D subtype

**Figure 3:**
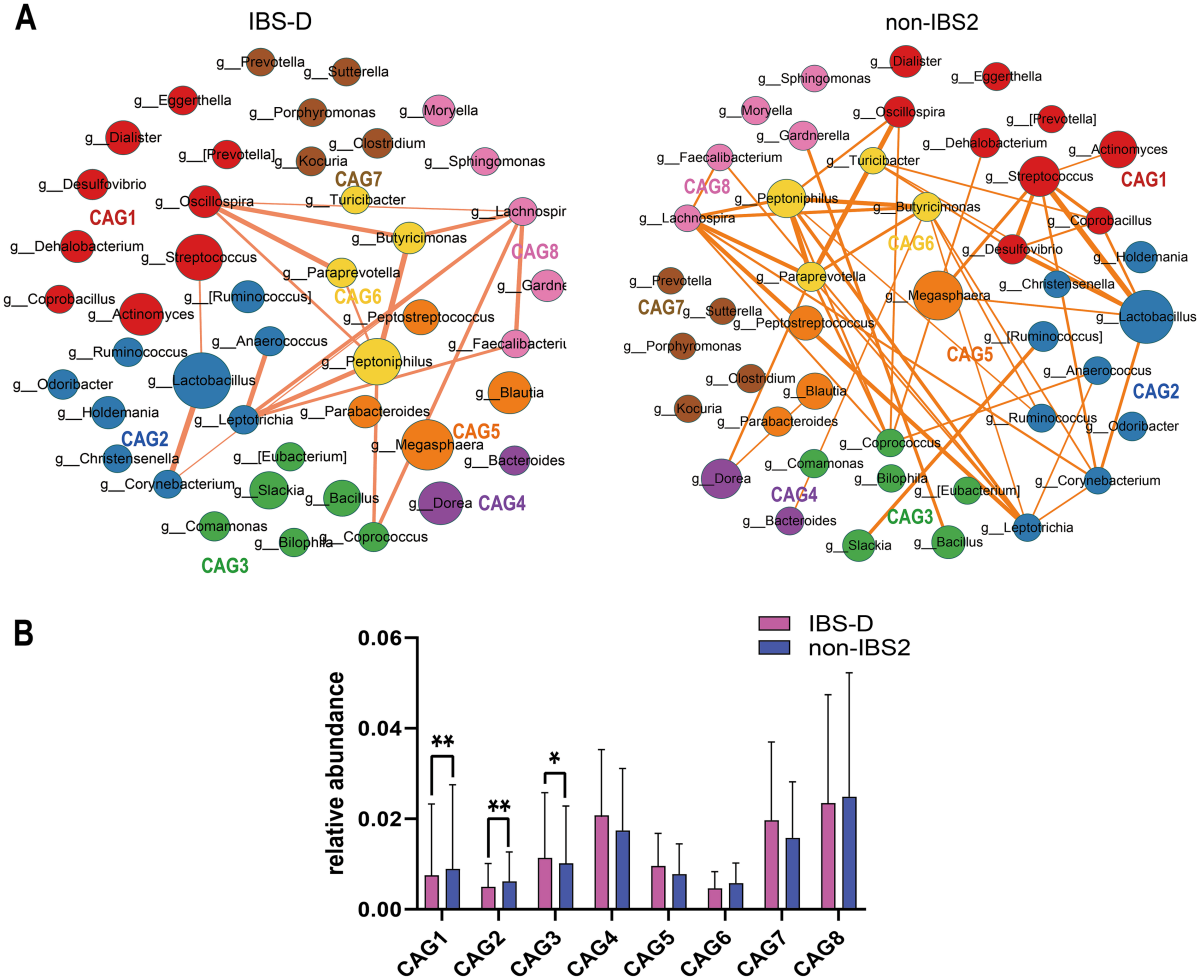
The species co-abundance network and Wilcoxon rank-sum test results for the average relative abundance of eight CAGs in the IBS-D group. **(A)** The genus-level co-abundance network. The node size indicates the average abundance of each genus, with different colors representing different CAGs. The lines between nodes depict their correlations, with line thickness reflecting the magnitude of the correlation coefficients. Only positive correlations with coefficients greater than 0.4 are displayed in the graph. **(B)** The Wilcoxon rank-sum test results for the average relative abundance of the eight CAGs, with red color representing the IBS-D group and purple color representing the non-IBS2 group. The results not reaching significance were not labeled with “ns” in the figure. ^*^p < 0.05, ^**^p < 0.01, ^***^p < 0.001, and ^****^p < 0.0001.

To investigate differences in bacterial genus co-abundance at the genus level within the IBS-D subtype, we categorized 44 enriched genera in IBS-D into eight CAGs, each containing between 2 to 9 genera (*p* < 0.05). Figure 3A illustrates the co-abundance networks of genera between the IBS and non-IBS2 groups. In the IBS-D group, 148 co-abundances were identified, compared to 92 in the non-IBS2 group. The enhancement of correlations between genera in the IBS-D group, compared to the non-IBS2 group, was primarily observed in CAG1, CAG2, CAG5, and CAG6. These involved *Oscillospira* and Streptococcus in CAG1; *Leptotrichia* in CAG2; *Megasphaera* and *Peptostreptococcus* in CAG5; and *Paraprevotella* and *Peptoniphilus* in CAG6. While decreased correlations were noted for *Coprococcus* in CAG3. Notably, the alterations in *Oscillospira* in CAG1; *Leptotrichia* in CAG2; *Coprococcus* in CAG3; and *Megasphaera* in CAG5 were particularly pronounced (Figure 3A). Based on the results of the Wilcoxon rank-sum test for the average relative abundances of the eight CAGs, in IBS-D patients, the average relative abundance of CAG3 significantly increased (*p* < 0.05), while those of CAG1 (*p* < 0.01) and CAG2 (*p* < 0.01) significantly decreased (Figure 3B).

### 3.4 The key findings on the co-abundance of gut microbiota in the IBS-U subtype

**Figure 4:**
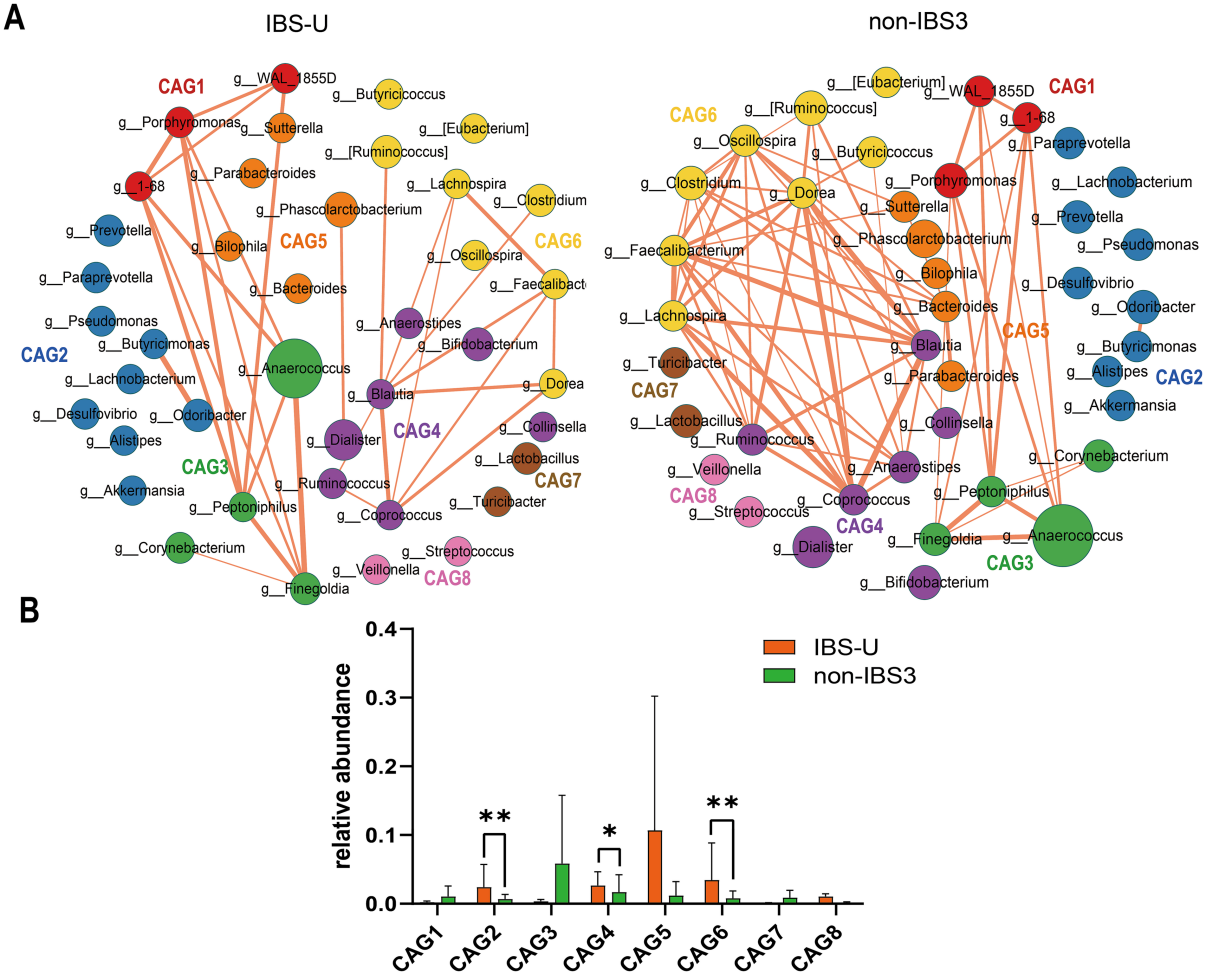
The species co-abundance network and Wilcoxon rank-sum test results for the average relative abundance of eight CAGs in the IBS-U group. **(A)** The genus-level co-abundance network. The node size indicating the average abundance of each genus and different colors representing different CAGs. The lines between nodes represent their correlations, with line thickness indicating the magnitude of the correlation coefficients. Only positive correlations with coefficients greater than 0.4 are shown in the graph. **(B)** The Wilcoxon rank-sum test results for the average relative abundance of the eight CAGs, with red representing the IBS-U group and green representing the non-IBS3 group. The results not reaching significance were not labeled with “ns” in the figure. ^*^p < 0.05, ^**^p < 0.01, ^***^p < 0.001, and ^****^p < 0.0001.

To investigate differences in bacterial genus co-abundance at the genus level within the IBS-U subtype, we categorized 40 enriched genera in IBS-U into eight CAGs, each containing between 2 to 9 genera (*p* < 0.05). Figure 4A depicts the co-abundance networks of genera between the IBS and non-IBS3 groups. A total of 58 co-abundances were identified in the IBS-U group, compared to 130 in the non-IBS group. In the analysis of genus-level co-abundance differences in the IBS-U subtype, significant co-abundance network differences were evident between the IBS-U group and the non-IBS group. Compared to the non-IBS3 group, the connectivity among bacteria in the IBS-U group primarily decreased in CAG4, CAG5, and CAG6. The involved genera include *Coprococcus*, *Blautia*, *Ruminococcus*, and *Anaerostipes* in CAG4; *Bacteroides* in CAG5; and *Clostridium*, *Lachnospira*, *Faecalibacterium*, *Dorea* and *Oscillospira* in CAG6 (Figure 4A).

According to the results of the Wilcoxon rank-sum test in Figure 4B (*p* < 0.05), for the average relative abundances of the eight CAGs, we observed that, compared to the non-IBS3 group, the average relative abundances of CAG2 (*p* < 0.01), CAG4 (*p* < 0.05), and CAG6 (*p* < 0.01) significantly increased in IBS-U patients. Notably, CAG2 includes core gut bacterial genera such as *Prevotella*, *Lachnobacterium*, and *Akkermansia* (Figure 4B). This suggests that the weakening of interactions among these beneficial bacteria could be a factor in the pathogenesis of IBS-U. These findings provide additional evidence of microbial composition differences between IBS-U patients and the non-IBS3 group, offering new insights into the pathogenesis of IBS-U.

### 3.5 Characteristic CAGs of different IBS subtypes were revealed based on random forest and SHAP analysis

**Figure 5:**
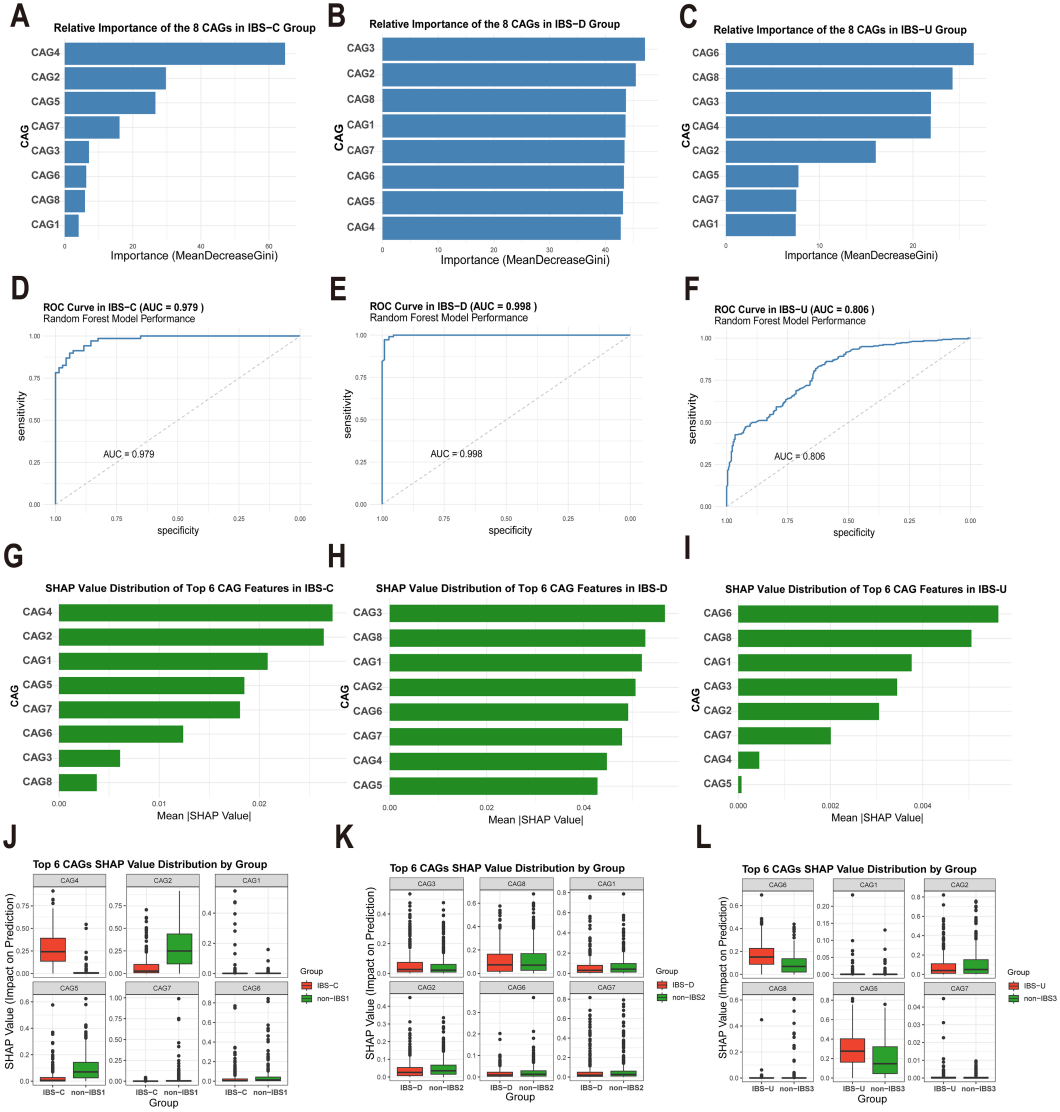
Analysis of the importance of gut microbiota characteristics and evaluation of model performance. **(A–C)** Relative importance of eight gut microbiota CAGs in different IBS subtypes, calculated based on the Gini index decrease method. **(D–F)** Receiver operating characteristic (ROC) curves and the area under the curve (AUC) of the random forest model for different IBS subtypes. **(G–I)** SHAP value distributions of the top six CAGs ranked by importance in different IBS subtypes. **(J–L)** Box plots of the SHAP value distributions of the top six CAGs grouped by different categories.

In the IBS-C group, CAG4 and CAG2 are of the most significant relative importance (Figure 5A). Similarly, the average SHAP values of CAG4 and CAG2 also rank among the top (Figure 5G). These results strongly suggest that CAG4 and CAG2 are highly likely to be the characteristic CAGs of IBS-C. For the IBS-D group, the CAG3 ranks first in terms of importance, and CAG2, CAG8, and CAG1 are also of relatively high importance (Figure 5B). The average SHAP values of these four CAGs are also very prominent (Figure 5H). This further confirms that CAG3, CAG2, CAG8, and CAG1 may be the characteristic CAGs of IBS-D, with CAG3 playing a particularly crucial role. In the IBS-U group, the CAG6 has the highest relative importance, and CAG3 and CAG4 are also relatively prominent (Figure 5C). The average SHAP value of CAG6 reaches the highest (Figure 5I), which further consolidates the important position of CAG6 and other CAGs in the IBS-U group.

The random forest model demonstrates excellent discriminative performance in different subtypes of IBS. The area under the curve (AUC) values of the IBS-C, IBS-D, and IBS-U groups are as high as 0.979, 0.998, and 0.806, respectively (Figures 5D–F). Figures 5J–L present the box-plots of the SHAP value distributions of the top six CAGs divided by groups. In the comparison between the IBS and non-IBS groups, significant inter-group differences in the SHAP value distributions of some CAGs can be observed. These differences provide strong evidence for screening the characteristic CAGs of IBS subtypes.

### 3.6 Analysis of the co-abundance differences in gut microbiota and metabolic pathways among different subtypes of IBS

**Figure 6:**
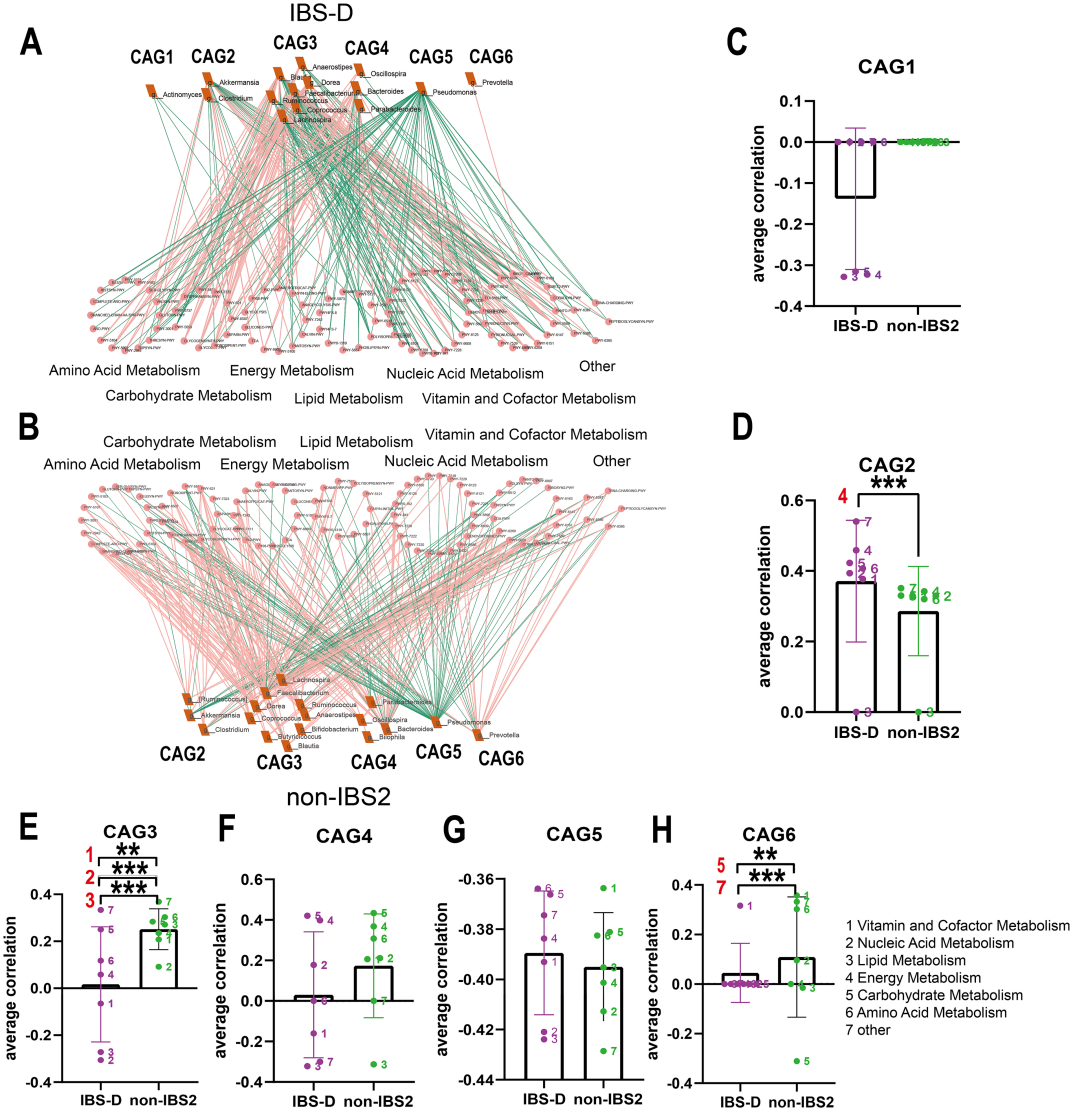
The co-abundance network and Wilcoxon rank-sum test results for the intestinal microbiota and metabolic pathways in IBS-D group. **(A,B)** The co-abundance network of the intestinal microbiota and metabolic pathways in IBS-D patients is depicted. Node size represents the average abundance of each genus and metabolic pathway, with circular nodes denoting metabolic pathways and rectangular nodes representing genera. Lines between nodes indicate correlations, with pink representing positive correlations and green representing negative correlations. The thickness of the lines indicates the absolute magnitude of the correlation coefficients. Only correlations with coefficients greater than 0.2 are displayed in the graph. **(C–H)** The Wilcoxon rank-sum test results for the average correlation coefficients between each CAG and different metabolic pathway categories in IBS-D. Different colors represent distinct metabolic pathway categories. The number to the left of the significance marker indicate the names of metabolically significant pathways. The results not reaching significance were not labeled with “ns” in the figure. ^*^p < 0.05, ^**^p < 0.01, ^***^p < 0.001, and ^****^p < 0.0001.

**Figure 7:**
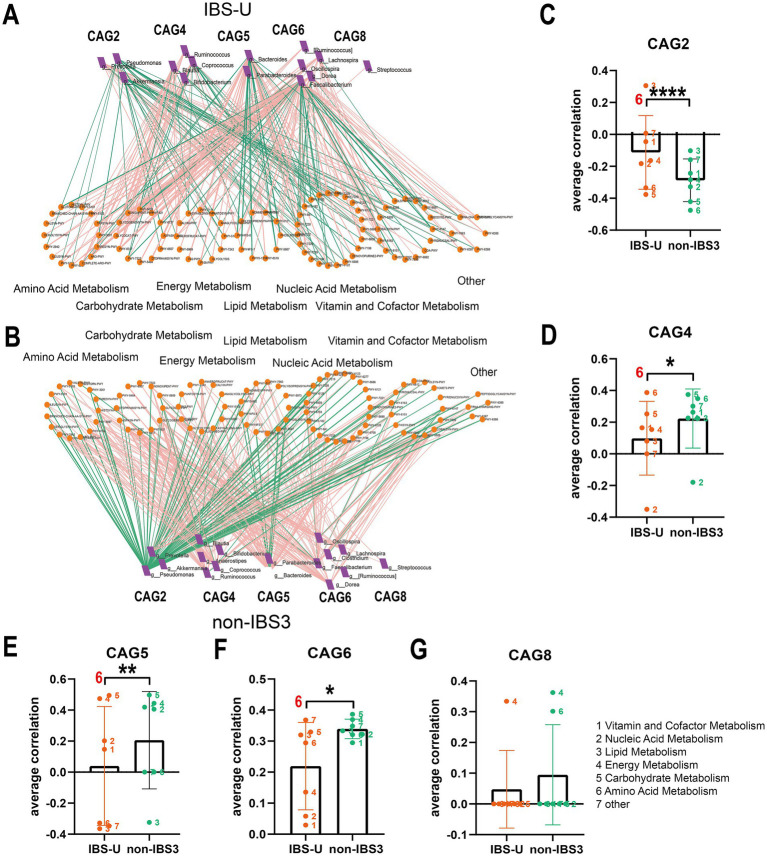
The co-abundance network and Wilcoxon rank-sum test results for the intestinal microbiota and metabolic pathways in IBS-U group. **(A,B)** The co-abundance network of the intestinal microbiota and metabolic pathways in IBS-U patients is shown. Node size represents the average abundance of each genus and metabolic pathway, with circular nodes denoting metabolic pathways and rectangular nodes representing genera. Lines between nodes indicate correlations, with pink representing positive correlations and green representing negative correlations. The thickness of the lines indicates the absolute magnitude of the correlation coefficients. Only correlations with coefficients greater than 0.2 are displayed in the graph. **(C–G)** The Wilcoxon rank-sum test results for the average correlation coefficients between each CAG and different metabolic pathway categories in IBS-U. Different colors represent distinct metabolic pathway categories. The number to the left of the significance marker indicate the names of metabolically significant pathways. The results not reaching significance were not labeled with “ns” in the figure. ^*^p < 0.05, ^**^p < 0.01, ^***^p < 0.001, and ^****^p < 0.0001.

To investigate whether changes in the abundance of certain bacteria lead to alterations in specific metabolic pathways, we analyzed the co-abundance of gut microbiota and metabolic pathways in IBS subtypes. Figures 6, 7 illustrates the network diagram of co-abundance between gut microbiota and metabolic pathways in IBS subtypes, along with the results of statistical analysis.

We calculated Spearman correlation coefficients between different IBS subtypes and bacteria associated with metabolic pathways, selecting co-abundances with correlation coefficients greater than 0.2 for further analysis. In IBS-C patients, no co-abundances with bacteria and pathways exhibited correlation coefficients greater than 0.2, with generally low correlation coefficients around 0.1, thus no further analysis was conducted.

In the IBS-D group, as depicted in Figures 6A,B, compared to the non-IBS2 group, there is a trend towards an increase in negative correlations between each CAG and various metabolic pathways. Statistical results reveal that the co-abundance of CAG2, CAG3, and CAG6 with metabolic pathways exhibits significant differences between the disease group and the non-IBS2 group (Figures 6C–H). Among these, the positive correlation between CAG2 genera and energy metabolic pathways exhibits increased strength (*p* < 0.001) (Figure 6D). The genera in CAG3 show a shift from positive to negative correlations with vitamin and cofactor metabolism (*p* < 0.01), nucleic acid metabolism (*p* < 0.001), and lipid metabolism (*p* < 0.001) (Figure 6E). The correlation coefficient of the genera in CAG6 with Carbohydrates metabolism decreases negatively (*p* < 0.01), and with other metabolic pathways, it decreases positively (*p* < 0.001) (Figure 6H). Some of these co-abundance groups have been previously reported; for instance, *Akkermansia*, belonging to CAG2, is known to produce acetate and butyrate to meet the host’s energy requirements (8), and the *Dorea* genus, part of the Firmicutes family *Clostridiales*, belonging to CAG3 is renowned for its fermentation metabolism utilizing a variety of substrates including glucose, fructose, lactose, and aromatic compounds (17).

In IBS-U patients, as depicted in Figures 7A,B, compared to the non-IBS3 group, there is a trend of either increase or decrease in the positive and negative correlation coefficients between each CAG and various metabolic pathways. Statistical results indicate that there are significant differences in the correlations between CAG2, CAG4, CAG5, and CAG6 and various metabolic pathways. Notably, CAG2, CAG4, CAG5, and CAG6 have all undergone significant changes in their correlation with the amino acid metabolism pathway. Specifically, the correlation coefficient between the amino acid metabolism pathway and CAG2 shows a negative decrease (*p* < 0.0001), with CAG4 it exhibits a positive increase (*p* < 0.05), with CAG5 it presents a negative increase (p < 0.01), and with CAG6 it displays a positive decrease (*p* < 0.05) (Figures 7C–G).

**Figure 8:**
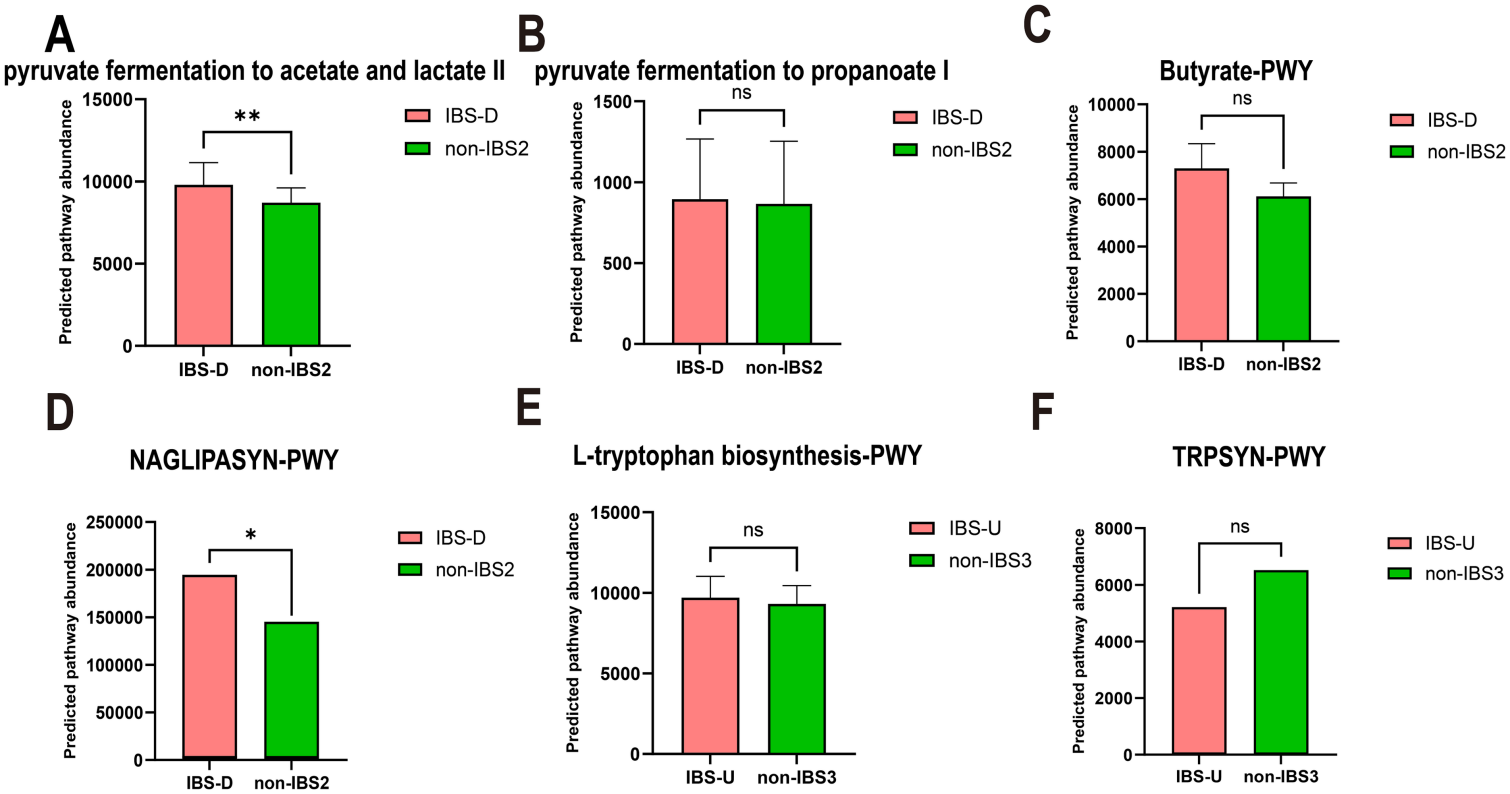
The paired *t*-test results for the predicted pathway abundance of SCFA, LPS-associated, and tryptophan metabolic pathways in IBS-D and IBS-U groups. **(A–C)** The predicted pathway abundance of the three short-chain fatty acid (acetate, propionate, and butyrate) metabolic pathways. **(D)** The predicted pathway abundance of the LPS-associated metabolic pathways. **(E,F)** The predicted pathway abundance of the tryptophan metabolism-associated metabolic pathways. ^*^p < 0.05 and ^**^p < 0.01, ns, no significant.

Accumulating evidence demonstrates that SCFAs, lipopolysaccharide (LPS), and tryptophan metabolism play crucial roles in the pathogenesis of IBS. Through analysis of differentially expressed metabolic pathways, this study revealed distinct metabolic disturbances in IBS-D patients: significant alterations were observed in both energy metabolism pathways related to bacterial SCFAs and lipid metabolism associated with LPS. Specifically, all three major SCFA biosynthesis pathways (acetate, propionate, and butyrate) exhibited upward trends, with acetate showing statistically significant changes while propionate and butyrate demonstrated consistent though non-significant trends (Figures 8A–C); concurrently, LPS-related metabolic pathways were significantly elevated (Figure 8D). In contrast, only amino acid metabolism pathways showed differences in IBS-U patients. Consequently, we primarily analyzed the predicted pathway abundance changes of tryptophan metabolic pathways (Figures 8E,F), which demonstrated a decreasing trend in IBS-U, but the difference was not statistically significant.

These findings suggest that alterations in the gut microbiota may lead to changes in metabolic pathways, subsequently influencing the progression of the disease and the manifestation of symptoms.

## 4 Discussion

The gut microbiome constitutes a complex ecosystem wherein microorganisms establish dynamic equilibrium through ecological interactions including nutrient competition, signaling molecule exchange, and immune evasion mechanisms, with existing studies conclusively demonstrating the prevalence of cross-feeding interactions within this system (18–21). Through comprehensive analyses of distinct IBS subtypes, our study not only revealed significant variations in microbial co-abundance patterns but also identified characteristic alterations in CAGs and associated metabolic pathways. These observed modifications likely reflect unique ecological interaction networks among microbial communities across different IBS subtypes.

We observed the key genera across subtypes of IBS. In the IBS-C group, the relative abundances of CAG2 and CAG4 increased significantly, mainly reflected in the genera *Clostridium* and *Bacteroides*. Moreover, in the random forest model, these two CAGs showed characteristic changes in the IBS-C group, indicating that they may be the key genera groups for IBS-C. A previous study also found an elevated number of *Clostridium* in IBS-C patients (22), which is consistent with the results of our study. However, the interactions among these genera with increased abundances were weakened. It is likely that the competitive effect reduced the interactions and metabolic products among the genera, thereby leading to hypo-osmotic constipation in the intestine. Nevertheless, a prior study reported a decrease in genera *Bacteroides* in IBS-C patients (23), while our study showed an increase. We speculate that the impact of genera *Bacteroides* on the organism may not be solely caused by changes in its abundance, but may also depend on its interaction patterns.

In addition, CAG3 with a reduced relative abundance contains various beneficial bacteria, such as *Faecalibacterium* with anti-inflammatory effects (24). *Blautia* which produces SCFAs (25) and *Bifidobacterium* and *Butyricicoccus* that have gut-protective effects (26, 27). In conclusion, constipation in IBS-C patients may be due to the reduction of gut microbial metabolic products, resulting in a hypo-osmotic state and weakened gut motility.

In the diarrhea-predominant IBS-D group, the top four CAGs predicted by the random forest model are CAG3, CAG2, CAG1, and CAG8. Among these CAGs, the relative abundance of CAG3 increased significantly, but the co-abundance network connectivity of the genera dominated by *Coprococcus* weakened. In contrast, the abundances of the other three CAGs decreased. This includes *Oscillospira*, which can promote cellulose degradation and the production of SCFAs (28), and *Leptotrichia*, which has pathogenic effects (29), however, the co-abundance network connectivity of CAG1 and CAG2 increased. This imbalance in the microbial community may have an impact on the host.

*Coprococcus* in CAG3 is a beneficial anti-inflammatory bacterium. The increase in its quantity in IBS-D patients may be an adaptive response (30). Although no association between *Megasphaera* and IBS has been previously reported, it can ferment various carbohydrates to produce acetate, propionate, and lactate (31). Its high abundance in the gut microbiome of colorectal cancer patients suggests a potential association with the disease (32), with significant variations observed across different populations (33). We hypothesize that overgrowth of these microbes or changes in specific co-abundance patterns lead to metabolic product accumulation, disrupting gut homeostasis, increasing intestinal permeability, and triggering diarrhea. Additionally, IBS-D may be associated with genetic factors and population-specific gut microbiome variations (34).

In the IBS-U group, CAG4 and CAG6 are enriched with a large number of dominant genera. According to the analysis of the random forest model, these two CAGs are suggested to be the characteristic CAGs of IBS-U. Their co-abundance network connectivity weakens while their relative abundances increase, which may represent the unique microbial community characteristics of IBS-U patients. Interestingly, the majority of these genera are reported to be beneficial bacteria, such as previous studies confirming the beneficial roles of *Bacteroides*, *Faecalibacterium*, and *Blautia* (20, 35). *Roseburia* can produce short-chain fatty acids to maintain gut luminal homeostasis (36). *Coprococcus* has also been confirmed as a beneficial bacterium in previous studies (37). Most of these are beneficial bacteria, yet their interaction is notably reduced, possibly due to a restrictive response to the increased abundance. CAG2 also contains *Prevotella*, *Lachnobacterium*, and *Akkermansia*, whose abundances remain elevated in the disease group. Therefore, the inconsistent changes in the abundance of beneficial bacteria and the weakening of their interactions may be the reason why the stool characteristics of patients with IBS-U remain normal. The weakening or inhibition of interactions among these microbial communities could impact the stability of the gut microbiome. However, further research is needed to elucidate the exact mechanisms involved.

We conducted a Venn diagram analysis of the microbiota in the CAGs of gut microbes of these three subtypes. The results showed that there were 37 genera shared by the three subtypes, indicating the presence of relatively stable gut microbiota in different IBS subtypes. There were 45, 47, and 43 genera shared between IBS-C and IBS-D, IBS-D and IBS-U, and IBS-C and IBS-U, respectively. In addition, IBS-C, IBS-D, and IBS-U had 8, 19, and 15 unique genera, respectively (Supplementary Figure 1). These data provide a reference for understanding the gut microbiota characteristics of different IBS subtypes.

In our correlation analysis between PICRUSt2 functional predictions and gut microbiota abundance, we observed that neither IBS-C patients nor the non-IBS1 group exhibited absolute correlation coefficients exceeding 0.2 between microbial taxa and metabolic pathways. We hypothesize this may reflect the extreme complexity of microbial interactions, where taxonomic abundance alone cannot determine metabolic outcomes, as inter-species competition, cross-feeding, and other ecological dynamics may fundamentally reshape metabolic pathway activities.

In patients with IBS-D and IBS-U, gut microbiota alterations and associated metabolic shifts critically influence disease progression: the weakened negative correlation between CAG2 [containing SCFA-producing *Lactobacillus* (38) and *Ruminococcus* (39)] and energy metabolism pathways (Figure 6D) reduces SCFA output, thereby impairing barrier function and promoting inflammation via increased permeability. Concurrently, CAG6’s diminished suppression of carbohydrate metabolism (Figure 6H), attributed to declines in protein-fermenting *Peptostreptococcus* (40), triggers aberrant protein fermentation, yielding epithelial-damaging metabolites such as ammonia, hydrogen sulfide (H₂S), and branched-chain fatty acids (BCFAs) (41), along with uncontrolled glycolysis. We hypothesize that this dysregulation leads to excessive carbohydrate breakdown with luminal SCFA accumulation, which elevates osmotic pressure and exacerbates diarrhea. This hypothesis is supported by the elevated predicted pathway abundance of SCFA metabolism observed in Figures 8A–C.

The metabolic association of CAG3 with vitamin, nucleic acid, and lipid metabolism shifted from positive to negative (Figure 6E), potentially impairing intestinal epithelial renewal through suppressed nucleic acid metabolism. Notably, IBS-D patients exhibited upregulated LPS-related pathways (Figure 8D), consistent with reported elevated plasma LPS levels (42) suggesting a pathogenic cascade: increased LPS abundance promotes Gram-negative bacterial proliferation, compromises barrier integrity, and induces systemic inflammation, ultimately driving lipid dysregulation and diarrhea. This integrates microbial-metabolic-inflammatory components in IBS-D pathophysiology. Additionally, reduced *Dorea* activity in CAG3 (43), may shift tryptophan metabolism toward neuroactive 5-HT production, aligning with visceral hypersensitivity in IBS-D.

In IBS-U patients, normal stool consistency likely reflects a dynamic equilibrium between gut microbiota and amino acid metabolism: CAG2 and CAG5 negatively regulate mucosal repair, whereas CAG4 and CAG6 synergistically activate protective branched-chain amino acid metabolism while suppressing harmful metabolites. Despite structural microbiota differences between IBS-U and non-IBS3 groups, compensatory adaptations in other bacterial populations or CAGs counteract these alterations to maintain intestinal homeostasis.

This study adopts a holistic approach to investigate gut microbial community interactions rather than focusing on individual microbes, providing comprehensive insights for population-based microbiome therapies. However, the exclusive reliance on the AGP database may introduce sampling bias, and metabolic pathway predictions generated by PICRUSt2 require experimental validation, highlighting the need for more diverse data sources. The cross-sectional study design limits causal interpretations regarding microbial alterations in IBS pathogenesis, necessitating future validation through germ-free animal models and fecal microbiota transplantation experiments. Although we identified significant correlations between microbial co-abundance networks and metabolic pathways, the specific biological mechanisms underlying these associations, which likely involve complex interactions across IBS subtypes, remain to be elucidated through further mechanistic investigations.

## 5 Conclusion

The study reveals significant differences in the co-abundance of gut microbiota among patients with different subtypes of IBS, suggesting that the onset of the disease may stem from the dysregulation of interactions among multiple microbial communities. The identification of key species in different subtypes and the mapping of metabolic pathway changes through species variations provide some hypotheses for the distinct symptoms of patients with different subtypes. These findings offer a new perspective on understanding the pathophysiological mechanisms of different IBS subtypes and imply the possibility of treating different IBS subtypes by modulating specific gut microbial communities.

## Data Availability

The original contributions presented in the study are included in the article/Supplementary material, further inquiries can be directed to the corresponding author.

## Footnotes

^1^ https://github.com/ivanvujkc/AGP_confounders

^2^ https://www.ncbi.nlm.nih.gov/

^3^ https://metacyc.org/

## References

[1] Oka P Parr H Barberio B Black CJ Savarino EV Ford AC. Global prevalence of irritable bowel syndrome according to Rome III or IV criteria: a systematic review and meta-analysis. Lancet Gastroenterol Hepatol. (2020) 5:908–17. doi: 10.1016/S2468-1253(20)30217-X, PMID: 32702295

[2] Lacy BE Mearin F Chang L Chey WD Lembo AJ Simren M. Bowel disorders. Gastroenterology. (2016) 150:1393–407. doi: 10.1053/j.gastro.2016.02.031 27144627

[3] Thompson JR. Is irritable bowel syndrome an infectious disease? World J Gastroenterol. (2016) 22:1331–4. doi: 10.3748/wjg.v22.i4.1331, PMID: 26819502 PMC4721968

[4] Chey WD Kurlander J Eswaran S. Irritable bowel syndrome: a clinical review. JAMA. (2015) 313:949. doi: 10.1001/jama.2015.0954, PMID: 25734736

[5] Takiishi T Fenero CIM Câmara NOS. Intestinal barrier and gut microbiota: shaping our immune responses throughout life. Tissue Barriers. (2017) 5:e1373208. doi: 10.1080/21688370.2017.1373208, PMID: 28956703 PMC5788425

[6] Su Q Tun HM Liu Q Yeoh YK Mak JWY Chan FK. Gut microbiome signatures reflect different subtypes of irritable bowel syndrome. Gut Microbes. (2023) 15:2157697. doi: 10.1080/19490976.2022.2157697, PMID: 36573834 PMC9809927

[7] Rowland I Gibson G Heinken A Scott K Swann J Thiele I. Gut microbiota functions: metabolism of nutrients and other food components. Eur J Nutr. (2018) 57:1–24. doi: 10.1007/s00394-017-1445-8, PMID: 28393285 PMC5847071

[8] Peña-Rodríguez M Vega-Magaña N García-Benavides L Zepeda-Nuño JS Gutierrez-Silerio GY González-Hernández LA. Butyrate administration strengthens the intestinal epithelium and improves intestinal dysbiosis in a cholestasis fibrosis model. J Appl Microbiol. (2022) 132:571–83. doi: 10.1111/jam.15135, PMID: 33982373

[9] Xiao L Liu Q Luo M Xiong L. Gut microbiota-derived metabolites in irritable bowel syndrome. Front Cell Infect Microbiol. (2021) 11:729346. doi: 10.3389/fcimb.2021.729346, PMID: 34631603 PMC8495119

[10] Fiorani M Del Vecchio LE Dargenio P Kaitsas F Rozera T Porcari S. Histamine-producing bacteria and their role in gastrointestinal disorders. Expert Rev Gastroenterol Hepatol. (2023) 17:709–18. doi: 10.1080/17474124.2023.2230865, PMID: 37394958

[11] Gui D-D Luo W Yan B-J Ren Z Tang Z-H Liu L-S. Effects of gut microbiota on atherosclerosis through hydrogen sulfide. Eur J Pharmacol. (2021) 896:173916. doi: 10.1016/j.ejphar.2021.173916, PMID: 33529724

[12] McDonald D Hyde E Debelius JW Morton JT Gonzalez A Ackermann G. American gut: an open platform for citizen science microbiome research. mSystems. (2018) 3:e00031-18. doi: 10.1128/mSystems.00031-18, PMID: 29795809 PMC5954204

[13] Vujkovic-Cvijin I Sklar J Jiang L Natarajan L Knight R Belkaid Y. Host variables confound gut microbiota studies of human disease. Nature. (2020) 587:448–54. doi: 10.1038/s41586-020-2881-9, PMID: 33149306 PMC7677204

[14] Bolyen E Rideout JR Dillon MR Bokulich NA Abnet CC Al-Ghalith GA. Reproducible, interactive, scalable and extensible microbiome data science using QIIME 2. Nat Biotechnol. (2019) 37:852–7. doi: 10.1038/s41587-019-0209-9, PMID: 31341288 PMC7015180

[15] Douglas GM Maffei VJ Zaneveld JR Yurgel SN Brown JR Taylor CM. PICRUSt2 for prediction of metagenome functions. Nat Biotechnol. (2020) 38:685–8. doi: 10.1038/s41587-020-0548-6, PMID: 32483366 PMC7365738

[16] Shannon P Markiel A Ozier O Baliga NS Wang JT Ramage D. Cytoscape: a software environment for integrated models of biomolecular interaction networks. Genome Res. (2003) 13:2498–504. doi: 10.1101/gr.1239303, PMID: 14597658 PMC403769

[17] Mechichi T Labat M Patel BKC Woo THS Thomas P Garcia J-L. *Clostridium methoxybenzovorans* sp. nov., a new aromatic o-demethylating homoacetogen from an olive mill wastewater treatment digester. Int J Syst Evol Microbiol. (1999) 49:1201–9. doi: 10.1099/00207713-49-3-1201, PMID: 10425780

[18] Chen L Collij V Jaeger M van den Munckhof ICL Vich Vila A Kurilshikov A. Gut microbial co-abundance networks show specificity in inflammatory bowel disease and obesity. Nat Commun. (2020) 11:4018. doi: 10.1038/s41467-020-17840-y, PMID: 32782301 PMC7419557

[19] Sinha AK Laursen MF Brinck JE Rybtke ML Hjørne AP Procházková N. Dietary fibre directs microbial tryptophan metabolism via metabolic interactions in the gut microbiota. Nat Microbiol. (2024) 9:1964–78. doi: 10.1038/s41564-024-01737-3, PMID: 38918470 PMC11306097

[20] Schirmer M Smeekens SP Vlamakis H Jaeger M Oosting M Franzosa EA. Linking the human gut microbiome to inflammatory cytokine production capacity. Cell. (2016) 167:1125–1136.e8. doi: 10.1016/j.cell.2016.10.020, PMID: 27814509 PMC5131922

[21] Bäumler AJ Sperandio V. Interactions between the microbiota and pathogenic bacteria in the gut. Nature. (2016) 535:85–93. doi: 10.1038/nature18849, PMID: 27383983 PMC5114849

[22] Lyra A Rinttilä T Nikkilä J Krogius-Kurikka L Kajander K Malinen E. Diarrhoea-predominant irritable bowel syndrome distinguishable by 16S rRNA gene phylotype quantifcation. World J Gastroenterol. (2009) 15:5936. doi: 10.3748/wjg.15.5936, PMID: 20014457 PMC2795180

[23] Hou Y Dong L Lu X Shi H Xu B Zhong W. Distinctions between fecal and intestinal mucosal microbiota in subgroups of irritable bowel syndrome. Dig Dis Sci. (2022) 67:5580–92. doi: 10.1007/s10620-022-07588-4, PMID: 35879512

[24] Zhou J Wei H Zhou A Xiao X Xie X Tang B. The gut microbiota participates in the effect of linaclotide in patients with irritable bowel syndrome with constipation (IBS-C): a multicenter, prospective, pre-post study. J Transl Med. (2024) 22:98. doi: 10.1186/s12967-024-04898-1, PMID: 38263117 PMC10807057

[25] Skrzydło-Radomańska B Prozorow-Król B Cichoż-Lach H Majsiak E Bierła JB Kanarek E. The effectiveness and safety of multi-strain probiotic preparation in patients with diarrhea-predominant irritable bowel syndrome: a randomized controlled study. Nutrients. (2021) 13:756. doi: 10.3390/nu13030756, PMID: 33652763 PMC7996889

[26] Yu S Chen J Zhao Y Liao X Chen Q Xie H. Association analysis of the gut microbiota in predicting outcomes for patients with acute ischemic stroke and H-type hypertension. Front Neurol. (2023) 14:1275460. doi: 10.3389/fneur.2023.1275460, PMID: 37954644 PMC10639143

[27] Laursen MF Sakanaka M von Burg N Mörbe U Andersen D Moll JM. *Bifidobacterium* species associated with breastfeeding produce aromatic lactic acids in the infant gut. Nat Microbiol. (2021) 6:1367–82. doi: 10.1038/s41564-021-00970-4, PMID: 34675385 PMC8556157

[28] Yang J Li Y Wen Z Liu W Meng L Huang H. Oscillospira—a candidate for the next-generation probiotics. Gut Microbes. (2021) 13:1987783. doi: 10.1080/19490976.2021.1987783, PMID: 34693878 PMC8547878

[29] He C Xie Y Zhu Y Zhuang K Huo L Yu Y. Probiotics modulate gastrointestinal microbiota after *Helicobacter pylori* eradication: a multicenter randomized double-blind placebo-controlled trial. Front Immunol. (2022) 13:1033063. doi: 10.3389/fimmu.2022.1033063, PMID: 36426355 PMC9679295

[30] Nikolova VL Smith MRB Hall LJ Cleare AJ Stone JM Young AH. Perturbations in gut microbiota composition in psychiatric disorders: a review and meta-analysis. JAMA Psychiatry. (2021) 78:1343–54. doi: 10.1001/jamapsychiatry.2021.2573, PMID: 34524405 PMC8444066

[31] Zhu X Hong G Li Y Yang P Cheng M Zhang L. Understanding of the site-specific microbial patterns towards accurate identification for patients with diarrhea-predominant irritable bowel syndrome. Microbiol Spectr. (2021) 9:e0125521. doi: 10.1128/Spectrum.01255-21, PMID: 34937163 PMC8694097

[32] Ingle AT Fortney NW Walters KA Donohue TJ Noguera DR. Mixed acid fermentation of carbohydrate-rich dairy manure hydrolysate. Front Bioeng Biotechnol. (2021) 9:724304. doi: 10.3389/fbioe.2021.724304, PMID: 34414173 PMC8370043

[33] Han S Wu W Da M Xu J Zhuang J Zhang L. Adequate lymph node assessments and investigation of gut microorganisms and microbial metabolites in colorectal cancer. Onco Targets Ther. (2020) 13:1893–906. doi: 10.2147/OTT.S242017, PMID: 32184624 PMC7061441

[34] Huang W Huang H-L Peng W Liu Y-D Zhou Y-L Xu H-M. Altered pattern of immunoglobulin A-targeted microbiota in inflammatory bowel disease after fecal transplantation. Front Microbiol. (2022) 13:873018. doi: 10.3389/fmicb.2022.873018, PMID: 35814647 PMC9257281

[35] Larsen N Bussolo De Souza C Krych L Barbosa Cahú T Wiese M Kot W. Potential of Pectins to beneficially modulate the gut microbiota depends on their structural properties. Front Microbiol. (2019) 10:223. doi: 10.3389/fmicb.2019.00223, PMID: 30828323 PMC6384267

[36] Song L Sun Q Zheng H Zhang Y Wang Y Liu S. *Roseburia hominis* alleviates neuroinflammation via short-chain fatty acids through histone deacetylase inhibition. Mol Nutr Food Res. (2022) 66:e2200164. doi: 10.1002/mnfr.202200164, PMID: 35819092 PMC9787297

[37] Sidhu SRK Kok CW Kunasegaran T Ramadas A. Effect of plant-based diets on gut microbiota: a systematic review of interventional studies. Nutrients. (2023) 15:1510. doi: 10.3390/nu15061510, PMID: 36986240 PMC10057430

[38] Zhang Y Zhou J Dong Z Li G Wang J Li Y. Effect of dietary copper on intestinal microbiota and antimicrobial resistance profiles of *Escherichia coli* in weaned piglets. Front Microbiol. (2019) 10:2808. doi: 10.3389/fmicb.2019.02808, PMID: 31921011 PMC6927916

[39] Verhaar BJH Hendriksen HMA de Leeuw FA Doorduijn AS van Leeuwenstijn M Teunissen CE. Gut microbiota composition is related to AD pathology. Front Immunol. (2021) 12:794519. doi: 10.3389/fimmu.2021.794519, PMID: 35173707 PMC8843078

[40] Chen GJ Russell JB. Fermentation of peptides and amino acids by a monensin-sensitive ruminal Peptostreptococcus. Appl Environ Microbiol. (1988) 54:2742–9. doi: 10.1128/aem.54.11.2742-2749.1988, PMID: 2975156 PMC204366

[41] Ashaolu TJ Ashaolu JO. Prebiotic peptides, their formation, fermentation in the gut, and health implications. Biotechnol Prog. (2021) 37:e3142. doi: 10.1002/btpr.3142, PMID: 33666376

[42] Gao J Xiong T Grabauskas G Owyang C. Mucosal serotonin reuptake transporter expression in irritable bowel syndrome is modulated by gut microbiota via mast cell-prostaglandin E2. Gastroenterology. (2022) 162:1962–1974.e6. doi: 10.1053/j.gastro.2022.02.016, PMID: 35167867 PMC9117493

[43] Zhang P Wang X Li S Cao X Zou J Fang Y. Metagenome-wide analysis uncovers gut microbial signatures and implicates taxon-specific functions in end-stage renal disease. Genome Biol. (2023) 24:226. doi: 10.1186/s13059-023-03056-y, PMID: 37828586 PMC10571392

