# Supplementary material for: Exploring the relationship between co-abundance of gut microbiota and novel metabolic pathways in different subtypes of irritable bowel syndrome: insights from the American Gut Project
Source: Front Med (Lausanne). 2025 Jul 22;12:1615717. doi: 10.3389/fmed.2025.1615717 (PMC12321851; doi:10.3389/fmed.2025.1615717)
Supplement: Supplementary file 1 [file Supplementary_file_1.docx]

Supplementary Figure 1


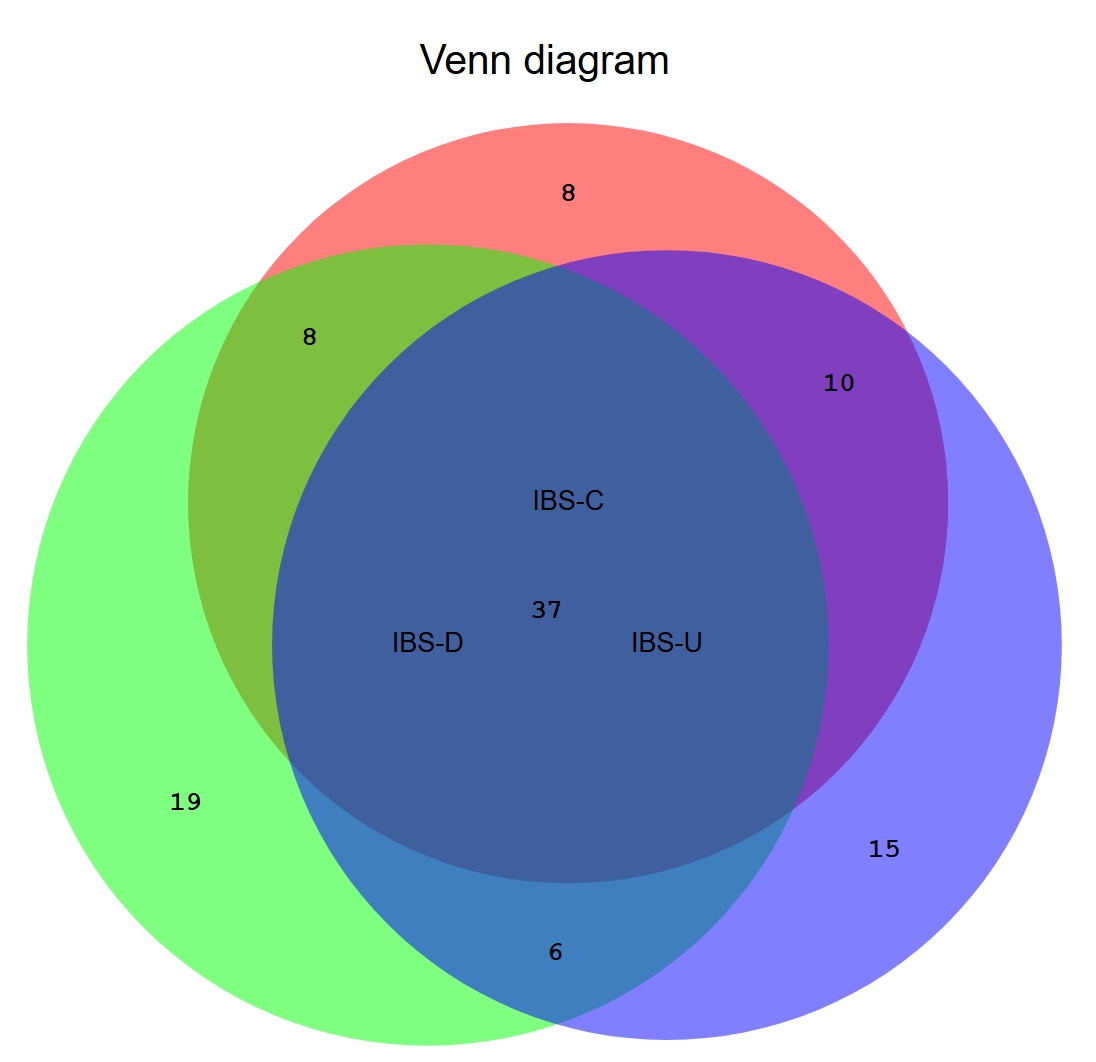


**Supplementary Fig.1.** Venn diagram of the common and specific gut microbiota genera among 24 different CAGs across the three subtypes of IBS. The red area in the Venn diagram represents the genera of bacteria present in the gut microbiota of patients with the IBS-C. The green area represents the genera of bacteria found in those with the IBS-D. The blue area represents the genera of bacteria in patients with the IBS-U. The overlapping regions indicate the genera of bacteria shared among different subtypes, while the non-overlapping parts represent the unique genera specific to each subtype.
